# A review of somatic single nucleotide variant calling algorithms for next-generation sequencing data

**DOI:** 10.1016/j.csbj.2018.01.003

**Published:** 2018-02-06

**Authors:** Chang Xu

**Affiliations:** Life Science Research and Foundation, Qiagen Sciences, Inc., 6951 Executive Way, Frederick, Maryland 21703, USA

**Keywords:** Variant calling, Somatic mutation, Unique molecular identifier, Low-frequency mutation, Benchmarking

## Abstract

Detection of somatic mutations holds great potential in cancer treatment and has been a very active research field in the past few years, especially since the breakthrough of the next-generation sequencing technology. A collection of variant calling pipelines have been developed with different underlying models, filters, input data requirements, and targeted applications. This review aims to enumerate these unique features of the state-of-the-art variant callers, in the hope to provide a practical guide for selecting the appropriate pipeline for specific applications. We will focus on the detection of somatic single nucleotide variants, ranging from traditional variant callers based on whole genome or exome sequencing of paired tumor-normal samples to recent low-frequency variant callers designed for targeted sequencing protocols with unique molecular identifiers. The variant callers have been extensively benchmarked with inconsistent performances across these studies. We will review the reference materials, datasets, and performance metrics that have been used in the benchmarking studies. In the end, we will discuss emerging trends and future directions of the variant calling algorithms.

## 1 Introduction

DNA mutation is the cause of cancer and a major focus of cancer research and treatment. Next-generation sequencing (NGS) is by far the most promising technology for *de novo* mutation detection, thanks to the huge amount of reads that modern sequencers can generate. Theoretically, all mutations regardless of the variant allele frequency (VAF) or genomic region can be *observed* given enough read depth. However, *calling* them with confidence is not trivial due to noise in the reads. Numerous bioinformatics tools have been developed to uncover mutations (variants) from sequencing reads, and such procedures typically consist of three components: read processing, mapping and alignment, and variant calling. First, low quality bases (usually near the 3’ end of reads) and exogenous sequences such as sequencing adapters are trimmed with read processing tools such as Cutadapt [1], NGS QC Toolkit [2], and FASTX-Toolkit. Some targeted sequencing protocols use PCR primers or unique molecular identifiers (UMI) during library preparation. In this case, custom-built read processing scripts may be required to trim and extract these oligonucleotides. Second, the cleaned reads are mapped to where they may come from in the reference genome, and then aligned base-by-base. Commonly used mapping and alignment tools include BWA [3], NovoAlign, and TMAP (for Ion Torrent reads) for DNA sequencing, and splice-aware aligners such as TopHat [4] and STAR [5] for RNA sequencing. PCR de-duplication, indel-realignment, and base quality recalibration can be performed in this step as outlined in the Genome Analysis Toolkits (GATK)’s best practice for variant calling [6], [7]. The last step, variant calling, is essentially a process of separating real variants from artifacts stemming from library preparation, sample enrichment, sequencing, and mapping/alignment. It has been a very active research field for years and plenty of variant callers have been developed, many freely available. The goal of this article is to review the state-of-the-art variant callers for somatic variants, in the hope to assist practitioners, especially non-bioinformaticians, to select the appropriate variant caller for their own applications.

The underlying assumptions are quite different for germline and somatic variant calling algorithms. Germline variants are expected to have 50 or 100% allele frequencies, therefore germline variant calling is essentially to determine which of the three genotypes, AA, AB, or BB, fit the data best [7], [8], [9], [10]. Most artifacts are present in low frequency and unlikely to cause trouble, because homozygous reference would be the most likely genotype in this case. But rejecting these artifacts is not as easy in somatic variant calling, because some real variants could also be present in very low frequencies in cases of impure sample, rare tumor subclone, or circulating DNA. Therefore, the biggest challenge of somatic variant calling is to disambiguate low-frequency variants from artifacts, which requires more sensitive statistical modeling and advanced error correction technology.

Genetic variants can be grouped into three categories by size: single nucleotide variant (SNV), insertion and deletion (indel), and structural variant (SV, including copy number variation, duplication, translocation, etc.). Very few variant callers are versatile enough to call all three because they require very different algorithms. For SNV and short indels (typically ≤ 10bp), the general strategy is to look for non-reference bases from the stack of reads that cover each position. Probabilistic modeling is critical here to infer the underlying genotype or evaluate the odds of variant versus artifacts. For structural variants and long indels, since the reads are too short to span over any variant, the focus is to locate the breakpoints based on the sudden change of read depth or patterns of misalignment with paired end reads. Split-reads and de-novo assembly methods are often used for SV and long indel detection.

In this review, we will focus on somatic SNV calling algorithms. We will review 46 publicly available somatic SNV callers that cover a wide spectrum of applications, in the hope to provide a practical guide for choosing the appropriate software. We will also explain the core algorithm of each variant caller and, if applicable, highlight the strengths and caveats. Germline-only callers, such as GATK UnifiedGenotyper/HaplotypeCaller, inGAP, and MAQ [6], [7], [11], [12] are not included in this review. Although UnifiedGenotyper and HaplotypeCaller have been used for somatic variant calling, their core algorithms are not designed for this task and perform poorly for low-frequency somatic variants, as stated in the GATK documentation and shown by independent studies [13], [14]. We will also exclude variant callers that are primarily used for pooled-samples such as CRISP and thunder [15], [16].

The article will be structured as follows. We will first describe the general workflow of somatic SNV calling in Section 2. Next, we will explain the core algorithms of individual variant callers and arrange them by the intended application in 3, 4, 5, 6. Each dedicated to one type of application. We will then discuss methods of evaluating variant calling performance and review recent progress in benchmarking studies in Section 7. Finally, we will summarize the research field and discuss future directions in Section 8.

## 2 General workflow of somatic SNV calling

### 2.1 Pre-processing

In general, variant callers consist of three components: pre-processing, variant evaluation, and post-filtering. The main purpose of pre-processing is to keep low-quality reads from entering the variant evaluation procedure. Read quality is typically measured by average base quality score, mapping quality score, and number of mismatches from the reference genome, etc. If the SNV caller follows a position-based strategy, which basically calls variant at each target position independently and is adopted by most SNV callers, a read can be included at one position and excluded at another, depending on the base quality scores at each individual position. Some variant callers such as Strelka [17] and VarDict [18] implement local indel realignments during pre-processing, resulting in better accuracy around indels. This can also be done using GATK IndelRealigner and BQSR (base quality score recalibration). PCR de-duplication is recommended in whole genome or whole exome sequencing data and can be performed with SAMtools or Picard tools. But it is not recommended in PCR-based amplicon sequencing applications where distinct DNA fragments can share the same genome coordinates. Also included in this step is downsampling during which a subset of reads are randomly selected to proceed to the next steps. Downsampling saves computation time and improves coverage uniformity if done at specific regions, but also makes the results non-deterministic.

### 2.2 Variant evaluation

Variant evaluation algorithm is the centerpiece of somatic variant callers and hence the focus of this review. Depending on the type of input data and the intended application, the algorithms can be summarized to four categories: matched tumor-normal variant calling, single-sample variant calling, UMI-based variant calling, and RNA-seq variant calling. Individual algorithms will be discussed in detail in 3, 4, 5, 6.

### 2.3 Post-filtering

Sequencing or alignment artifacts may appear to have strong read evidence and trick the statistical model to pass them as real variants. Most variant callers apply a set of filters to identify these artifacts and hence improve the specificity. Strand bias filter, for example, catches artifacts whose reads are only or dominantly observed on one strand, a common error in Illumina reads [19], [20]. Strand bias filters rely on the Fisher's exact test to identify imbalanced strand distribution. A number of filters focus on repetitive regions such as homopolymer, microsatellite, or low complexity regions, which are known to cause false calls due to increased alignment and sequencing errors [21], [22]. Hard filters are used in most variant callers, either completely rejecting variants in certain regions or relying on empirical hard thresholds [23].

## 3 Matched tumor-normal variant calling

### 3.1 Description of algorithms

The majority of current somatic variant callers are designed to analyze matched tumor-normal samples from the same patient. The fundamental idea is to identify potential variants using the tumor and distinguish somatic variants from germline and loss of heterozygosity (LOH) variants using the matched normal sample.

The heuristic approaches, adopted by VarScan2, qSNP, Shimmer, RADIA, SOAPsnv, and VarDict [9], [18], [24], [25], [26], [27], identify potential variants whose supporting reads meet certain thresholds and then apply statistical tests or *ad hoc* rules to isolate somatic variants. For example, VarScan2 requires at least two supporting reads and 8*%* VAF for a potential SNV (adjustable by users). Other callers have similar thresholds in their algorithms, which are typically set above the noise level of general NGS data and expected to filter out low-level artifacts. Next, the potential SNV sites are analyzed in the matched normal to filter out non-somatic variants. VarScan2, Shimmer, SOAPsnv, and VarDict apply Fisher's exact test on the 2 × 2 contingency table of read counts (reference vs. non-reference and tumor vs. normal). A small p-value indicates that non-reference reads are disproportionately distributed in the pair of samples and therefore suggests somatic variant. qSNP and RADIA apply sets of heuristic rules to label somatic variants that are sufficiently observed in tumor but weakly or not observed in normal. If RNA-seq data from the same patient are available, RADIA will include the gene expression data in an integrated analysis to further reduce false positives.

Joint genotype analysis, adopted by SomaticSniper, FaSD-somatic, SAMtools, JointSNVMix2, Virmid, SNVSniffer, Seurat, and CaVEMan [8], [28], [29], [30], [31], [32], [33], [34], assumes diploidy in both tumor and normal and evaluates the likelihood of the joint genotypes. Variant calling becomes a natural corollary of the genotye inference. At the core of these algorithms is the posterior probability of the joint genotypes, calculated by Bayes' rule, i.e.,

$$P(G_{T},G_{N}|D_{T},D_{N})=\frac{P(D_{T},D_{N}|G_{T},G_{N})P(G_{T},G_{N})}{\sum_{g_{T},g_{N}\in G}P(D_{T},D_{N}|g_{T},g_{N})P(g_{T},g_{N})},$$

where *G*_*T*_,*G*_*N*_ are genotypes of tumor and normal and *D*_*T*_,*D*_*N*_ are reads in tumor and normal. The prior genotype probability *P*(*G*_*T*_,*G*_*N*_) may depend on genome-wide SNP rate, somatic mutation rate, Ti-Tv ratio, etc. The joint likelihood of data, *P*(*D*_*T*_,*D*_*N*_|*G*_*T*_,*G*_*N*_), can be calculated with Binomial probability by viewing bases covering a site as independent Bernoulli trials whose success probability depends on the genotype and sequencing error rate. Once the joint genotypes are inferred, somatic variant calling follows naturally. SomaticSniper and FaSD-somatic summarize the evidence of somatic mutation by a “somatic score”, which is essentially the log-transformed probability of tumor and normal having the same genotype. The score is given by − 10log _10_*P*(*G*_*T*_ = *G*_*N*_|*D*_*T*_,*D*_*N*_), where *G*_*T*_ ∈{*AA, AC, AG, AT, CC, CG, CT, GG, GT, TT*}. Sites with higher somatic score are more likely to have different genotype in tumor and normal and are identified as potential somatic variants subject to post-filters. SAMtools follows the same strategy, but instead of posterior probability, uses log-likelihood ratio as the somatic score. JointSNVMix2, Virmid, and SNVSniffer collapse the ten explicit genotypes into AA, AB, and BB (A being the reference and B being non-reference) and therefore reduce the joint genotypes to a 3 × 3 table. Somatic variant calling is equivalent to calculating *P*(*somatic*) = *P*(*AA, AB*) + *P*(*AA, BB*), the probability of homozygous-reference in normal and heterozygous or homozygous-non-reference in tumor. Specifically, JointSNVMix2 applies a hierarchical Bayesian model to estimate joint genotype probabilities. Virmid views tumor as a mixture of normal tissues and somatic mutations and provides a joint estimation of the joint genotypes and proportion of normal tissue in tumor. SNVsniffer takes a hybrid approach of heuristic and joint genotype analysis. High-confidence somatic variants from heuristic analysis are reported directly and low-confidence variants require further examination of joint genotype probability estimation. Seurat combines AB and BB into one category (both called “non-reference”) and calculates the probability of reference in normal and non-reference in tumor. CaVEMan applies an expectation-maximization algorithm to estimate the genotype probabilities.

The diploidy assumption may be overly simplified for tumor due to the presence of rare heterogeneous subclones within a tumor sample. To uncover variants in complex tumor genomes, especially in rare subclones, some variant callers abandon the diploidy assumption and model joint allele frequencies (*f*_*T*_,*f*_*N*_) instead of joint genotypes (*G*_*T*_,*G*_*N*_). The allele frequency analysis approach is taken by Strelka, MuTect, LoFreq, EBCall, deepSNV, LoLoPicker, and MuSE [17], [35], [36], [37], [38], [39], [40]. Strelka's core algorithm consists of two steps. First, the posterior probabilities of VAFs in tumor and normal, noted as *P*(*f*_*T*_,*f*_*N*_|*D*_*T*_,*D*_*N*_), are estimated based proportions of non-reference bases. Second, somatic variant probability is calculated as the probability that VAFs differ in the pair of samples and that the normal sample's genotype is homozygous reference, i.e., *P*(*f*_*T*_≠*f*_*N*_|*D*_*T*_,*D*_*N*_)*P*(*ref*,*ref*|*D*_*N*_). MuTect formulates somatic variant calling as two model selection problems. In tumor, two models are evaluated and compared: the wild-type model **M**^0^ that assumes all non-reference reads come from technical artifacts and the mutation model **M**^*f*^ that assumes that variant allele is present at an unknown frequency *f*. A log-likelihood ratio (“LOD score”) is computed to select the better fitted model. At potential mutation sites (high LOD score), another model selection is performed in normal to compare the wide-type model **M**^0^ and the heterozygous model **M**^0.5^. If **M**^0^ is strongly preferred than **M**^0.5^, the variant is labeled as somatic. LoFreq, EBCall, deepSNV, and LoLoPicker formulate variant calling as a hypothesis testing problem in which the null hypothesis is wild-type, alternative hypothesis is variant, and the test statistic is the observed non-reference reads *n*_*T*_. LoFreq views each base as an independent Bernoulli trial with distinct “success” probability, where success is defined as non-reference and the probability is determined by the quality score. In this setting, *n*_*T*_ follows a Poisson-binomial distribution and the p-value can be calculated as the probability of observing more non-reference reads than *n*_*T*_. Because somatic variants are known to be enriched in certain hot-spots, sequence contexts, and non-coding regions, EBCall, deepSNV, and LoLoPicker estimate *site-specific* error rates and therefore allow distinct and more accurate detection limit at each site. In particular, deepSNV and LoLoPicker are designed to call low-frequency variants with targeted sequencing data. EBCall and deepSNV do not rely on quality scores to infer error rates, but assume that at each target position, the error rate is a random variable and follows a Beta distribution. Under the null hypothesis, *n*_*T*_ follows a Beta-binomial distribution and the p-value is calculated accordingly. In EBCall, Beta distribution parameters are obtained from sequencing of other independent control samples. In deepSNV, the parameters are estimated using tumor and normal samples of the current experiment. Similar strategy is adopted by LoLoPicker with an important modification that site-specific error rates are assumed to be fixed values. The site-specific error rates are particularly useful for variant calling with low quality samples such as formalin-fixed and paraffin-embedded (FFPE) samples, where error rates are higher and more uneven from site to site compared to fresh samples. However, the estimation of site-specific error rates requires sequencing of large number of samples, which is not always feasible. MuSE views somatic SNVs as the result of DNA revolution and models the process with a continuous-time Markov process with a state space of A, T, G, C. The equilibrium frequency of the non-reference allele is compared to a sample-specific threshold obtained from independent public datasets.

Haplotype-based strategy (as opposed to the mainstream position-based strategy) is widely adopted by structural variant callers in which reads need to be assembled to reconstruct long variants. It is also a powerful strategy for SNV detection and used by Platypus, HapMuC, LocHap, FreeBayes, and MuTect2 [35], [41], [42], [43], [44]. These algorithms locally assemble reads in a region and generate candidate haplotypes that may be represented by de Bruijn-like graphs. The likelihood of each haplotype is estimated by aligning each individual read to the haplotype and counting the read support. Haplotype-based variant callers have advantage in variant-dense region because they do not rely on the local alignment which is error-prone in the difficult regions. Haplotype-based callers also provide additional information about the co-existence of variants. For haplotype-based callers, indel re-alignment is no longer because the original local alignment information is discarded and reads are assembled and re-aligned.

Machine learning methods have been very successful in classification, and variant calling is essentially a classification problem. MutationSeq, SomaticSeq, SNooPer, and BAYSIC [45], [46], [47], [48] are representative variant callers that apply machine learning methods. MutationSeq extracts relevant features on each site and trains four classifiers (random forest, Bayesian adaptive regression tree, support vector machine, and logistic regression) based on the features and a set of “ground truth” somatic variants. The trained classifiers are then tested on naive datasets. SNooPer trains a random forest classifier and is designed to work on low-coverage data. SomaticSeq follows the same supervised training-testing procedure but differs from MutationSeq or SNooPer in two aspects. First, it uses adaptive boosting algorithm for classification. Second, it is an ensemble variant caller that requires the union of variant calls from other software (MuTect, SomaticSniper, VarScan2, JointSNVMix2, and VarDict) as a starting point and then applies its own classifier to remove false positives. BAYSIC is also an ensemble variant caller and applies an unsupervised latent class model to combine multiple calls.

### 3.2 Practical considerations on choosing the appropriate algorithm

The choice of variant caller largely depends on the what type of variants is of interest. For example, while all of the aforementioned variant callers report SNVs, only some offer indel and/or SV detection. Therefore, it would be convenient to choose the more versatile variant callers if indels or SV are of interest. The desired VAF is another important factor. In general, variant callers based on joint genotype analysis (SomaticSniper, FaSD-somatic, JointSNVMix2, Virmid, SNVSniffer, and Seurat) are designed for low-coverage data (WGS, WES, or targeted sequencing with low depth) and not sensitive enough to detect low-frequency variants, because the diploidy assumption in tumor implies that real variants' allele frequency should be around 0.5 or 1.0. To call low-frequency variants, especially with high-coverage targeted sequencing data, one should choose variant callers that model allele frequencies directly (Strelka, MuTect, LoFreq, EBCall, deepSNV, LoLoPicker, and MuSE). This important distinction has been emphasized in past reports [17], [35] and demonstrated in independent benchmark studies. For example, Xu et al. [13] showed that the sensitivity and specificity of SomaticSniper are much lower than Strelka and MuTect for variants with VAF *≤* 8*%* variants. But for variants with VAF ≥ 18*%*, SomaticSniper achieved comparable accuracy. Heuristic analysis-based callers can also achieve good accuracy with low-frequency variants if the thresholds are carefully chosen, as demonstrated in [49] (1% variant calling with VarDict) and [50] (< 5% variant calling with VarScan2).

The choice of variant caller also depends on the available data. Most callers take the standard input: aligned reads (BAM format) of matched tumor-normal samples, but some require additional information. For example, LoLoPicker requires a cohort of control samples to obtain the site-specific error rates. LocHap requires a list of SNVs called by other algorithms to perform haplotype analysis. SomaticSeq requires variant calls from a number of somatic variant callers and offers a dockerized version to save users' trouble of installing and running many different pipelines. In addition, most of the variant callers are developed for Illumina sequencing data, although some claim to be compatible with other sequencing technologies. Specialized callers are available and preferred for non-Illumina reads, such as Torrent Variant Caller (TVC) for Ion Torrent sequencing data and PoreSeq [51] for nanopore sequencing data (tumor-only).

The tumor-normal variant callers reviewed in this article are listed in Table 1.

**Table 1:** List of tumor-normal somatic SNV callers sorted in alphabetical order. For each variant caller, the types of variants that are reported (column 2), whether single-sample input is allowed (column 3), and a high-level summary of the core algorithm (column 4) are provided. The variant callers and their core algorithms are explained in detail in Section 3.

Variant caller	Type of variant	Single-sample mode	Type of core algorithm
BAYSIC [48]	SNV	No	Machine learning (ensemble caller)
CaVEMan [34]	SNV	No	Joint genotype analysis
deepSNV [38]	SNV	No	Allele frequency analysis
EBCall [37]	SNV, indel	No	Allele frequency analysis
FaSD-somatic [31]	SNV	Yes	Joint genotype analysis
FreeBayes [44]	SNV, indel	Yes	Haplotype analysis
HapMuC [42]	SNV, indel	Yes	Haplotype analysis
JointSNVMix2 [30]	SNV	No	Joint genotype analysis
LocHap [43]	SNV, indel	No	Haplotype analysis
LoFreq [36]	SNV, indel	Yes	Allele frequency analysis
LoLoPicker [39]	SNV	No	Allele frequency analysis
MutationSeq [45]	SNV	No	Machine learning
MuSE [40]	SNV	No	Markov chain model
MuTect [35]	SNV	Yes	Allele frequency analysis
SAMtools [8]	SNV, indel	Yes	Joint genotype analysis
Platypus [41]	SNV, indel, SV	Yes	Haplotype analysis
qSNP [24]	SNV	No	Heuristic threshold
RADIA [26]	SNV	No	Heuristic threshold
Seurat [33]	SNV, indel, SV	No	Joint genotype analysis
Shimmer [25]	SNV, indel	No	Heuristic threshold
SNooPer [47]	SNV, indel	Yes	Machine learning
SNVSniffer [32]	SNV, indel	Yes	Joint genotype analysis
SOAPsnv [27]	SNV	No	Heuristic threshold
SomaticSeq [46]	SNV	No	Machine learning (ensemble caller)
SomaticSniper [28]	SNV	No	Joint genotype analysis
Strelka [17]	SNV, indel	No	Allele frequency analysis
TVC [97]	SNV, indel, SV	Yes	Ion Torrent specific
VarDict [18]	SNV, indel, SV	Yes	Heuristic threshold
VarScan2 [9]	SNV, indel	Yes	Heuristic threshold
Virmid [29]	SNV	No	Joint genotype analysis

## 4 Single-sample variant calling

In practice, matched normal samples are not always available, so variant calling base only on tumor is desired. Some tumor-normal variant callers, such as MuTect and VarDict, accept single sample as input (Table 1). In addition, several algorithms are dedicated to perform single-sample variant calling. These algorithms include SNVMix2, shearwater, SPLINTER, SNVer, OutLyzer, Pisces, ISOWN, SomVarIUS, and SiNVICT [52], [53], [54], [55], [56], [57], [58], [59], [60] and fall into two categories.

SNVMix2, Shearwater, SPLINTER, SNVer, OutLyzer, and Pisces report all variants without distinguishing somatic and germline. SNVMix2’s, like JointSNVMix2 and Virmid, infers the posterior probability of each genotype. Shearwater is similar to LoLoPicker and requires a cohort of control samples to estimate site-specific error rates. The original Shearwater relies on a Bayesian model and uses Bayes factors to call variants. The newer version, ShearwaterML, takes a frequentist modeling approach and uses likelihood ratio test for variant calling. SPLINTER and SNVer are originally designed for SNP calling in population but also work on individual patients. SPLINTER generates run-specific error models with pooled samples to detect low-frequency variants. SNVer tests if the VAF is above certain threshold based on Binomial distribution. OutLyzer uses an outlier identification method (Thompson Tau test) to measure the background noise level and then call variants with non-reference bases above that level. Pisces is tuned for amplicon sequencing data. SNV calling in Pisces is determined by a q-score based on reference and non-reference read counts and a Poisson model. Pisces does not consider variants with VAF below 1*%* or variants in low-coverage area (minimum 10x coverage required).

ISOWN, SomVarIUS, and SiNVICT offer somatic-germline classification without the matched normal. ISOWN relies on MuTect2 (single-sample mode) to call all the variants in the sample and then uses supervised machine learning algorithms to train a somatic-germline classifier. The classification is based on a set of features including membership of databases for somatic (COSMIC) and germline mutations (ExAC and dbSNP), VAF, clinical impact of the mutation, sequence context, etc. SomVarIUS assesses the upper bound of the probability that all non-reference reads come from sequencing errors using Chernoff's equation. The upper bound is used to distinguish real variants from sequencing errors. The somatic-germline classification is performed by estimating the VAF distribution of heterozygous germline SNPs and labeling any variants whose VAF is on the left tail of that distribution as somatic. Furthermore, SomVarIUS uses matched RNA-seq data to help detect variants that are less supported by DNA data. SiNVICT is designed to call low-frequency variants in circulating tumor DNA (ctDNA). Poisson models are used to identify potential variants and to test if the VAF is significantly lower than 0.5. Importantly, SiNVICT can run on the same tumor at multiple stages and perform time-series analysis, which is particularly useful to understand how tumor evolves.

Single-sample sequencing often occurs in retrospective studies where old FFPE tumor tissues have no matched normal sample. Among these tools, OutLyzer, ISOWN, and SomVarIUS emphasized the application on FFPE samples and showed performance data in their publications. Another major application is low-frequency variant calling in targeted panel sequencing. OutLyzer, Pisces, ISOWN, SomVarIUS, SiNVICT have been validated in targeted sequencing applications. In fact, Pisces and SiNVICT are specifically designed for amplicon sequencing data.

The single-sample variant callers reviewed in this article are listed in Table 2.

**Table 2:** List of single-sample somatic and germline SNV callers sorted in alphabetical order. For each variant caller, the types of variants that are reported (column 2), whether somatic variants are distinguished from germline variants (column 3), applications reported in the original publication (column 4), and a high-level summary of the core algorithm (column 5) are presented. The variant callers and their core algorithms are explained in detail in Section 4.

Variant caller	Type of variant	Somatic-germline classification	Reported application	Type of core algorithm
ISOWN [34]	SNV	Yes	Deep sequencing, FFPE samples	Supervised learning
OutLyzer [56]	SNV	No	Deep sequencing, FFPE samples	Noise level estimation
Pisces [57]	SNV, indel	Yes	Deep sequencing	Poisson model on read count
PoreSeq [51]	SNV, indel	No	Low-coverage nanopore data	Nanopore specific
Shearwater [53]	SNV	No	Deep sequencing	Noise level estimation
SiNVICT [60]	SNV, indel	No	Deep sequencing; cfDNA	Poisson model on read count
SNVer [55]	SNV, indel	No	Deep sequencing	Allele frequency analysis
SNVMix2 [52]	SNV	No	WGS, WES	Genotype analysis
SomVarIUS [59]	SNV, indel	Yes	WES; FFPE samples	Noise level estimation
SPLINTER [54]	SNV, indel	No	Deep sequencing	Noise level estimation

## 5 UMI-based variant calling

### 5.1 UMI technology and variant calling

**Fig. 1:**
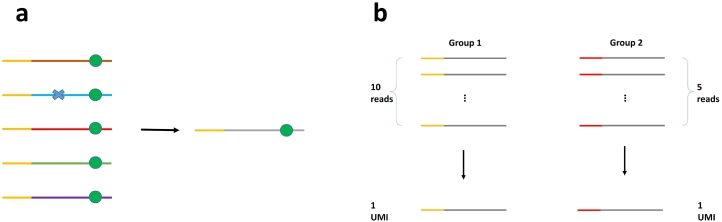
(a) Building a consensus read from a UMI group. Errors (blue cross) are corrected and real mutations (green circle) are preserved. Yellow segment indicates UMI sequence. (b) Reducing amplification bias by counting UMIs instead of reads.

Low-frequency variants (VAF ≤ 5*%*) are often confounded by sequencing errors that exist at a rate of 0.01–0.1 per base on Illumina platforms [61], [62] and DNA polymerase errors during PCR enrichment, which occur at a lower rate. To correct these errors, unique molecular identifiers (UMIs, or molecular barcodes) have been used in recent targeted sequencing protocols and shown to significantly improve the accuracy of low-frequency variant detection [63], [64], [65], [66], [67], [68], [69], [70], [71]. UMIs are attached to the original DNA fragments through ligation or primer extension and then carried through to enrichment and sequencing. The UMI sequences are retrieved from sequencing reads, allowing each read to be traced back to the original molecule. Through base-call consensus and UMI counting, most sequencing and DNA polymerase errors can be corrected and amplification bias can be reduced (Fig. 1). Ultimately, the detection limit of UMI-based variant calling is determined by the first-cycle PCR errors that propagate through amplification. Multiple studies [49], [65], [72] have shown that UMI-based protocols generate reads with lower error rate (after consensus), resulting in remarkably higher specificity compared to raw-reads-based variant calling.

Currently, three UMI-based variant callers are available in public domain: DeepSNVMiner, MAGERI, and smCounter [49], [73], [74]. DeepSNVMiner first generates an initial list of variants using SAMTools calmd and then selects the high-confidence variants with strong UMI support. MAGERI builds a consensus read for each UMI group of reads and takes a similar Beta-binomial modeling approach as EBCall [37]. The difference is, rather than estimating the sequencing error distribution, MAGERI estimates the DNA polymerase error (i.e. first-cycle PCR error) distributions using external data. In addition, MAGERI assumes a universal Beta distribution across all sites rather than site-specific error rates. smCounter implicitly generates the position-by-position consensus base call and calculates the posterior probability of variant by jointly considering PCR and sequencing errors. Both DeepSNVMiner and MAGERI are end-to-end pipelines that have built-in functions of UMI extraction, mapping and alignment, and variant calling. smCounter is a stand-alone variant caller that takes binary alignment map (BAM) data as input. Recently, TVC has released a plug-in for handling UMI-tagged Ion Torrent reads.

An alternative approach for UMI-based variant calling is to first construct consensus reads from UMI families by majority voting or weighted scoring at each base, then apply the raw-reads-based callers on the consensus read set [65], [75]. This approach is attractive because it is conceptually simple and easy to implement with open-source UMI tools such as Fgbio [76]. However, as pointed out in [49], [74], the caveat of this two-stage approach is that the base quality scores of the consensus reads are unlikely to be compatible with the downstream caller's error model.

### 5.2 Ultra low-frequency variants and duplex sequencing

Recent developments in early cancer diagnostics have raised the demand for detecting circulating tumor DNA (ctDNA). In these applications, calling 0.1% or lower variants is often required given the minute amount of ctDNA in blood. To reach such low detection limit, high-fidelity DNA polymerase must be used in sample enrichment to minimize first-cycle PCR errors. In addition, duplex sequencing that tags double-strand DNA and allows the reads from the two strands to be matched has been implemented to further reduce error rates [64], [77]. For a duplex UMI pair, first-cycle PCR errors can be identified as they most likely will occur in only one strand. The probability of the same DNA polymerase error occurring on both strands is theoretically the square of the standard error rate, which is typically lower than 10^−8^, depending on the fidelity of the enzyme. However, in current duplex sequencing protocols, only about 20% of the UMIs can be matched to the other strand due to insufficient ligation efficiency. Therefore, variant calling for duplex sequencing data has to rely on both singular and duplex UMIs. Two such algorithms are MEGARI and iDES [72].

### 5.3 UMI clustering

A common problem with UMI-based protocols is the sequencing or PCR errors within the UMI sequence, leading to “fake” UMIs. The common solution is to merge UMIs within short edit distance (typically 1 or 2) if they have different read counts. For example, Peng et al. [65] clusters a UMI to its “parent” UMI that is within one edit distance and has more than six times more reads. Kou et al. [78] merge two UMIs within 2-base difference based on binomial probability. MAGERI clusters two UMIs that differ by one or two substitutions and whose read counts differ by 20- or 400-fold. Smith et al. [79] developed UMI-tools that implemented network-based UMI clustering methods. Evaluation of these clustering algorithms using real data would be valuable but no such study has been published, to the author's knowledge.

The UMI-based variant callers reviewed in this article are listed in Table 3.

**Table 3:** List of UMI-based somatic and germline SNV callers sorted in alphabetical order. For each variant caller, the types of variants that are reported (column 2), whether a complete workflow including UMI handling (extraction, consensus, clustering), read processing, and mapping/alignment is provided (column 3), whether duplex sequencing data are supported (column 4), the library preparation and sequencing protocol companion to the caller (column 5), and the detection of limit reported in the original publication (column 6) are presented. The variant callers and their core algorithms are explained in detail in Section 5.

Variant caller	Type of variant	Complete workflow	Duplex sequencing data	Companion protocol	Detection limit (original paper)
DeepSNVMiner [73]	SNV, indel	Yes	No	Unspecified	0.1%
iDES [72]	SNV, indel	Yes	Yes	CARP-Seq	0.00025–0.025%
MAGERI [74]	SNV, indel	Yes	Yes	Multiple protocols	0.1%
smCounter [49]	SNV, indel	No	No	QIAseq targeted DNA-seq	1%

## 6 RNA-seq variant calling

The main purposes of RNA-seq experiments are gene expression analysis or gene fusion detection. As a side product, variant calling can be performed on the complementary DNA (cDNA) reads, which may contain more information than genomic DNA. For example, low-frequency variants may not be adequately observed in genomic DNA but enjoy high read support in RNA if the corresponding genes are highly expressed. On the other hand, RNA-seq variant calling will have lower accuracy compared to DNA, because of 1) increased alignment errors near splicing junctions; 2) increased error rate during reverse transcription; 3) failure to observe variants in non- or low- expressed genes and poor read depth uniformity due to variation in expression levels; and 4) RNA editing sites being confused as DNA mutations.

Currently there are at least variant callers that accept RNA-seq data: RADIA, Seurat, VarDict, VarScan2, SNPiR, and eSNV-detect [9], [18], [26], [33], [80], [81]. RADIA and Seurat do not analyze RNA-seq data alone, but integrate RNA-seq with matched tumor-normal DNA from the same patient to improve the accuracy. RADIA showed that real variants with weak evidence in DNA may be rescued by RNA data and that false positives that escaped DNA filters may be caught by additional filters on RNA data. VarDict and VarScan2 are well known DNA variant callers but also compatible with RNA-seq data without matched DNA samples. SNPiR and eSNV-detect are dedicated RNA-seq variant callers. SNPiR maps RNA reads to the reference genome, uses GATK to call variants on the aligned reads, identifies spurious calls near splice junctions and homopolymers, and cross-checks with RNA-editing database to filter known RNA-editing sites. eSNV-detect takes BAM files from two aligners and uses SAMtools for variant calling. Variants identified by both aligners are called with high confidence and one aligner low confidence.

The RNA-seq variant callers reviewed in this article are listed in Table 4.

**Table 4:** List of RNA-seq somatic and germline SNV callers sorted in alphabetical order. For each variant caller, the types of variants that are reported (column 2), whether DNA-RNA integrated analysis is performed (column 3), whether the tool is exclusively for RNA-seq variant calling (column 4), and whether a complete workflow including RNA-seq read mapping, variant calling, and filtering is provided (column 6) are presented. The variant callers and their core algorithms are explained in detail in Section 6.

Variant caller	Type of variant	Integrated analysis	Dedicated to RNA-seq	Complete workflow
eSNV-detect [81]	SNV	No	Yes	No
RADIA [26]	SNV	Yes	No	No
Seurat [33]	SNV, indel	Yes	No	No
SNPiR [80]	SNV	No	Yes	Yes
VarDict [18]	SNV, indel, SV	No	No	No
VarScan2 [9]	SNV, indel	No	No	No

## 7 Benchmarking variant calling performance

### 7.1 Benchmarking studies

Although most variant callers were published with benchmarking results against other mainstream pipelines of their time, the claimed performance may not be replicated on independent datasets. A number of independent studies to benchmark and compare various somatic variant callers have been published [13], [14], [50], [82], [83], [84], [85], but inconsistent performance data and contradicting rankings of the variant callers were reported. The inconsistency of benchmarking results is due to two reasons. First, most variant callers need to be fine-tuned to achieve the expected accuracy on a naive dataset, yet the optimal parameter values are unknown to the tester. In this case, applying the default values seems a reasonable solution and indeed a common practice in benchmarking studies. For example, Cai et al. [84] applied default settings in comparing four tumor-normal callers. Sandmann et al. [14] used default settings except for VAF threshold. Kroigard et al. [85] applied default settings for when benchmarking on exome-sequencing data and adjusted parameters for targeted sequencing data. Second, some variant callers were original designed for certain types of applications and then published without extensive validation on a wide range of datasets, so their performance may drop in some occasions.

Competition-based benchmarking studies such as the DREAM mutation calling challenge [86] and the PrecisionFDA Truth Challenge (germline variants) leave the parameter-tuning work to the variant caller developers. The participants submit their own pipelines that are fine-tuned to training sets provided by the organizer, and winners are determined based on independent test sets.

### 7.2 Data and materials

Three types of materials are commonly used to generate data for benchmarking studies: synthetic reads, reference standards, and real tumor samples. Synthetic reads with configured variations are traditionally generated by read simulators with built-in or user-supplied error models such as ART [87] and SeqMaker [88]. Alternatively, hybrid datasets featuring real reads and simulated variants at arbitrary VAFs can be generated using BAMSurgeon [86]. Synthetic reads can be generated in large scale, at virtually no cost, and most importantly, contain known variants. However, synthetic data alone are generally considered inadequate because the artifacts and variations in real sequencing reads are more complex than the simulated data.

Reference standards can be sequenced to generate real validation data, but have long faced the challenge of lacking the ground truth variant set. In 2014, Genome in a Bottle Consortium (GIAB) published a high-confidence variant set for NA12878 cell line using multiple sequencing technologies and several combinations of aligners and variant callers [89], [90]. The variant set has been updated periodically since first published and high-confidence variant sets for more reference samples have been released by the GIAB Consortium. Several studies generated virtual tumors or tumor-normal pairs by mixing two GIAB cell lines at different ratios [13], [49], [65]. The downsides of this approach are 1) GIAB samples are from healthy donors and do not have cancer mutations; 2) the mixture sample contains variants with fixed allele frequencies, while real tumors contain a spectrum of VAFs; and 3) the GIAB high-confidence variant set is not 100% accurate and fails to cover some difficult regions. Somatic reference standards are also available, such as COLO829/COLO829BL cell lines from paired melanoma/normal samples [91], but it is unclear about the completeness of the final variant set given the low coverage of sequencing runs (less than 150X).

Real tumors-normal samples or ctDNA would be ideal for the validation of somatic variant callers if all the variants in the sample are known a priori. While this is rarely the case, orthogonal technologies such as Sanger sequencing and digital PCR can be used to validate the called variants (although Sanger sequencing has a limited VAF). But these methods are often expensive or laborious and, most importantly, not suitable for the discovery of *de novo* variants. Therefore they can be used to confirm whether the variants being called are real, but cannot verify what variants are missed.

In summary, each of the data sources has merit but also lacks important features for benchmarking studies. Cancer cell lines with complete, high-confidence variant set would better meet the need and greatly benefit the research community.

### 7.3 Performance metrics

For SNV callers, commonly used performance metrics include sensitivity, specificity, false positive rate, positive predictive value (PPV), false discovery rate (FDR), and F-score. The definitions are given in Table 5. Since mutations are very rare in genome and most variant callers have low false positive rate, specificities are often represented by long fractions such as 99.999…%. For easier interpretation, false positive rate represented as the number of false calls per megabase pair (Mbp^−1^) is often preferred over specificity [13], [40], [49]. ROC curve is also very commonly used in benchmarking studies to visually illustrate the sensitivity-specificity trade-off. The area under the ROC curve (AUC), a fraction between 0 and 1, measures the overall accuracy under a range of variant calling thresholds. AUC should only serve as a supplementary metric because it does not inform the accuracy under optimal or default threshold. As suggested in [92], confidence intervals should be reported to acknowledge the inherent sampling variation of these metrics.

**Table 5:** Definition of variant calling performance metrics. TP, TN, FP, FN are true positive, true negative, false positive, false negative respectively.

Metric	Synonym	Formula	Relation with other metrics
Sensitivity	Recall	$\frac{TP}{TP+FN}$	
Specificity		$\frac{TN}{TN+FP}$	
False positive rate (FPR)		$\frac{FP}{TN+FP}$	1 - specificity
Positive predictive value (PPV)	Precision	$\frac{TP}{TP+FP}$	
False discovery rate (FDR)		$\frac{FP}{TP+FP}$	1 - PPV
F-score	*F*_1_ score	$2\times \frac{\text{sensitivity}\times \text{PPV}}{\text{sensitivity}+\text{PPV}}$	harmonic mean of sensitivity and PPV

**Fig. 2:**
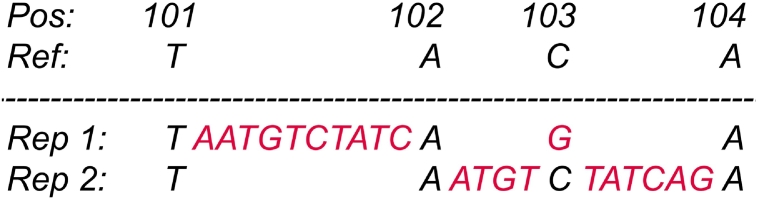
Illustration of a complex variant at position 101: TACA > TAATGTCTATCAGA being represented in two combinations of simple SNV and indels. Representation one: insertion at 101: T > TAATGTCTATC and SNV at 103: G > C. Representation two: insertions at 102: A > AATGT and 103: C > CTATCAG.

Complex variants consisting of several nearby SNVs and indels may be reported in seemingly different representations (Fig. 2), making it complicated to compare variant files generated by different variant callers. Variant normalization and comparison tools like vt normalize [93] and vcfeval in RTG Tools [94] are useful for this task.

## 8 Summary and outlook

Variant calling algorithms have been evolving and improving in the past years. The underlying models are getting more and more complex in order to describe the physical process of NGS experiments and to model different types of artifacts. For example, traditional tumor-normal SNV callers did not face the pressing need of detecting low-frequency variants, so their algorithms are still focused on inferring the most probable genotype. “modern” somatic SNV callers are expected to confidently call mutations that are barely above the noise level, therefore they ditched the diploidy assumption and modeled the VAF directly. Recently, UMI-based variant callers developed novel algorithms to use correct sequencing artifacts and to model the first-cycle PCR artifacts. From the algorithmic perspective, we have observed a trend of position-based variant callers being upgraded to haplotype-based variant callers (e.g. UnifiedGenotyper to HaplotypeCaller, MuTect to MuTect2) due to the inherent advantage in indels, structural variants, complex variants, and generally in high mutation loading regions. Looking at the bigger picture, an emerging trend is the use of deep learning algorithms for variant calling. Traditional model-based variant callers rely heavily on ad-hoc filters to reduce false calls because artifacts are generated in a very complex way that is beyond simple modeling. As a result, a variant caller often contains dozens of parameters and some of them can only be understood or safely tuned by the developers, hampering the practical utility. Deep neural networks (DNN) have recently been applied to variant calling with superior performance and more importantly, the trained model can be easily applied to other datasets with consistent performance. DNN-based algorithm has been demonstrated by the winner of PrecisionFDA Truth Challenge (germline variant calling), DeepVariant [95], and applied to somatic variant calling [96].

Variant callers are also evolving to accommodate new sequencing and library construction technologies. Traditional variant callers rely on base quality scores to wrestle with sequencing errors, but the base quality scores may not faithfully reflect the probability of base-calling errors. With the implementation of UMI and duplex sequencing, sequencing errors can be effectively eliminated given enough read replications. The new challenge is DNA polymerase errors that an order of magnitude lower than sequencing errors. New variant callers like smCounter, MAGERI, and iDES have been developed to handle UMI data, and existing variant callers such as TVC have been upgraded with plug-ins for UMI. These tools have greatly pushed the limit of detection down to 1%, 0.1% or lower. Looking forward, emerging technologies such as bi-modal DNA- and RNA-seq and single-cell sequencing may require new bioinformatics tools for variant calling.

Limited by time and budget, current benchmarking studies often fail to provide a wide range of datasets and fine-tune the variant calling parameters for optimal performance, resulting in biased and sometimes contradicting conclusions. Competition-based benchmarking studies like DREAM Mutation Calling Challenge and PrecisionFDA Truth Challenge provide several representative datasets, include a larger pool of variant callers (some under development and unpublished), allow participants to set the pipeline parameters, and evaluate the performance using consistent metrics. These features make the competition-based benchmarking results more credible. However, for somatic variant callers, independent and unbiased benchmarking is still limited by the lack of good validation datasets. Datasets used in recent benchmarking studies include synthetic and semi-synthetic reads, reference standards including GIAB samples and other cell lines, and real tumor-normal pairs. None of these are perfect validation data for reasons discussed above. We believe that the research community will benefit greatly from a collection of real cancer genomes that are deep sequenced to generate high-confidence GIAB-like variant sets.
